# Laparoscopic resection for gastric schwannoma larger than 30 mm with long-term outcomes

**DOI:** 10.1186/s12893-023-02190-5

**Published:** 2023-09-19

**Authors:** Xuetong Jiang, Mingzuo Zhao, Jianqiang Wu, Yang Ding, Jian Wang

**Affiliations:** 1 Department of Gastrointestinal Surgery, the Affiliated Suqian Hospital of Xuzhou Medical University (Suqian Hospital of Nanjing Drum Tower Hospital Group), No. 138 Huanghe South Road, Suqian, 223800 China; 2 Department of Pathology, the Affiliated Suqian Hospital of Xuzhou Medical University (Suqian Hospital of Nanjing Drum Tower Hospital Group), No. 138 Huanghe South Road, Suqian, 223800 China

**Keywords:** Schwannoma, Stomach, Laparoscopy surgery

## Abstract

**Background and aims:**

Laparoscopic resection has been reported as effective and safe for gastric schwannoma (GS) in the form of case reports. However, study on laparoscopic surgery in patients with GS larger than 30 mm has been rarely reported. To this end, the present study aimed to evaluate the safety and efficacy of laparoscopic resection for the treatment of GS larger than 30 mm and its long-term outcomes.

**Methods:**

This is a retrospective case series study of patients with GS larger than 30 mm who underwent laparoscopic resection at our hospital between January 2014 and December 2020. Clinical pathology, surgical and follow-up data were collected and analyzed.

**Results:**

A total of 10 patients with a mean age of 51.6 years were included. Seven tumors were located in gastric body, 2 in antrum and 1 in fundus. Laparoscopic gastric wedge resection was performed in 7 patients, while laparoscopic gastric local resection was performed in 3 patients. All patients achieved complete resection. The mean operation time was 112.6 ± 34.3 min, and the mean postoperative hospital stay was 13.8 ± 5.1 days. Postoperative gastroplegia occurred in 2 patients and was treated with conservative therapy. No recurrence, metastasis or residue was found during the follow-up of mean 45.1 months.

**Conclusions:**

Laparoscopic resection is a safe and effective method for treating GS larger than 30 mm with favorable long-term follow-up outcomes. Laparoscopic resection may be considered as the first-line treatment for GS larger than 30 mm.

## Introduction

Schwannomas are neurogenic tumors arising from the sheath of schwann cells, most commonly found in spinal cord, central nervous system and peripheral nerves of extremities [1]. Gastrointestinal schwannomas are extremely rare, and are most commonly found in stomach. Gastric schwannoma (GS) accounts for about 4% of benign gastric tumors and 0.2% of all gastric tumors [2], which often found by esophagogastroduodenoscopy. Patients with GS are usually asymptomatic, but some symptomatic patients may present as gastrointestinal bleeding, abdominal pain and abdominal mass [3, 4]. Although GS is considered a benign tumor and has a very rare potential for malignant transformation, it is still difficulty to be distinguished from gastrointestinal stromal tumor (GIST) and leiomyoma before surgery [5].

The correct diagnosis is determined by postoperative pathology and immunohistochemistry [6]. Resection is the main method of curative treatment. Laparoscopy, laparotomy and endoscopic resection are all feasible methods. The treatment depends on the location and size of the tumor as well as the willingness of the patient. Recently, endoscopic resection, such as endoscopic full-thickness resection and endoscopic submucosal excavation, has been reported as an effective and minimally invasive treatment for GS [7, 8], with tumor size as an important factor in its selection. Zhai et al. [9] reported that endoscopic resection could be used as the first-line treatment for patients with GS less than 30 mm. In the case of a large mass, especially a size larger than 30 mm, the tumor should be divided into several parts to be completely removed under endoscopy, which increases the risk of tumor spread. Surgical resection may be the best choice for GS larger than 30 mm. Laparoscopic resection has been reported as effective and safe for GS in the form of case reports [10, 11]. No study on laparoscopic surgery in patients with GS larger than 30 mm has been reported. Thus, the aim of this study was to evaluate the role of laparoscopic resection in treating GS larger than 30 mm and its long-term outcomes.

## Materials and methods

### Study design and patients

This retrospective case series study was conducted at a single center and approved by the Ethics Committee of the Affiliated Suqian Hospital of Xuzhou Medical University. The medical records of patients between January 2014 and December 2020 were reviewed. Written informed consents were obtained from all patients before the laparoscopic procedure.

The inclusion criteria were as follows: (1) patients were diagnosed as GS confirmed by postoperative pathology; (2) patients having undergone laparoscopic surgery; (3) patients with a mass larger than 30 mm in diameter and the tumor size was determined by CT or endoscopic examination preoperatively and confirmed by postoperative pathology; (4) patients with complete medical records. Clinical data, including demographic characteristics, clinical symptoms, preoperative gastroscopy or endoscopic ultrasound (EUS) and imaging contrast data, operation details, adverse events, postoperative pathological and immunohistochemical data and follow-up were collected from databases as well as outpatient and inpatient medical records. All clinical data were analyzed by 2 independent researchers. For any disagreement in the review work, a discussion was conducted until an agreement was reached.

### Laparoscopic procedures

**Fig. 1:**
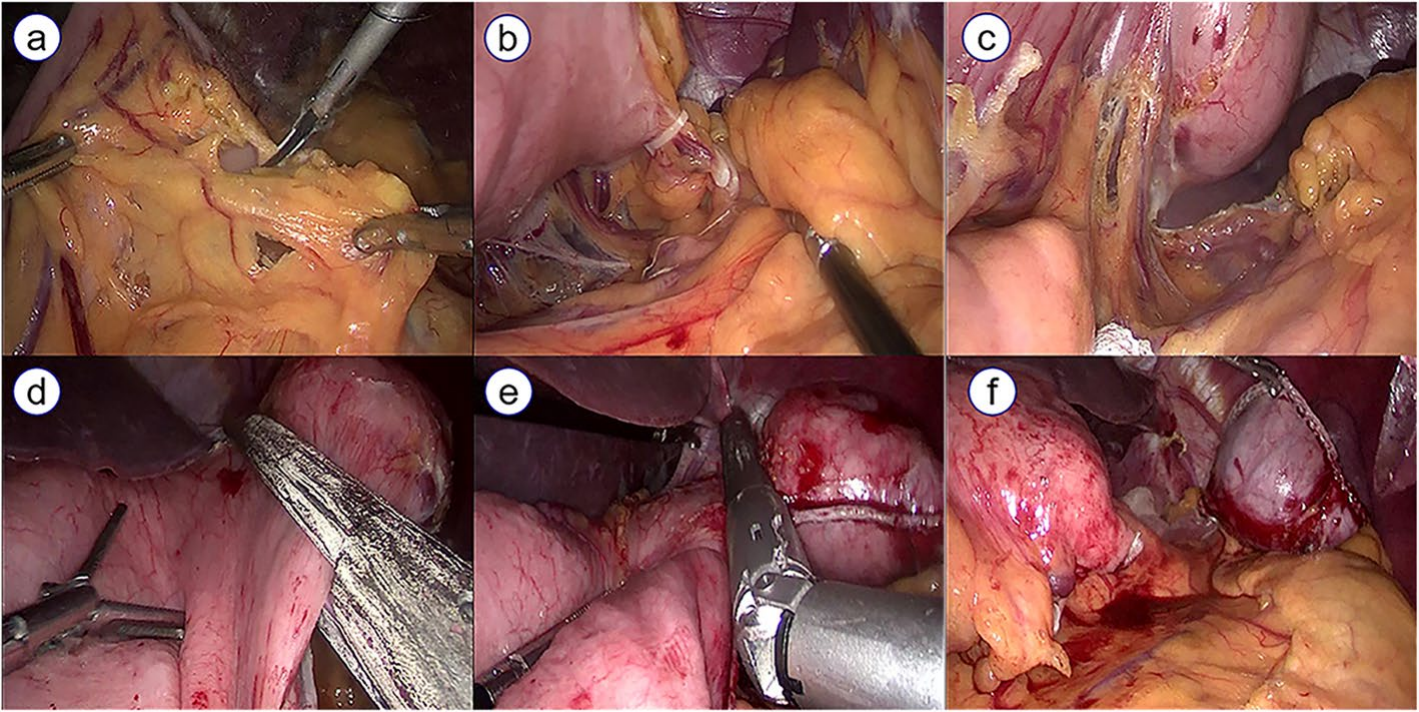
Laparoscopic gastric wedge resection procedures. **a** separated gastric mesangium; **b** ligated vasa brevia; **c** separated posterior gastric artery; **d** remove the tumor by laparoscopic linear cutting stapler; **e** another stapler to complete gastric wedge resection; **f** resection tumor specimen

All patients were subjected to general anesthesia and placed in supine position with head high, feet low and legs divided. The surgeon was positioned to the patient’s left, and the assistant was positioned to the patient’s right. Routine umbilical puncture was used to establish pneumoperitoneum with a pressure of 12–15 mmHg. After insertion of the laparoscopy through umbilical trocar, four trocars were placed in upper abdominal wall to make a V-shape together. After the abdominal organs were explored, the mesangium of the gastric tumor was separated by an ultrasonic knife, and the blood vessels near the tumor were ligated according to the intraoperative needs. The surgical method was selected according to the location and size of the tumor. Laparoscopic gastric wedge resection (Fig. 1) was the main treatment method for surgically removing GS. The tumor was removed with a margin of around 20 mm by a laparoscopic linear cutting stapler. For a tumor near the pylorus or cardia, laparoscopic gastric local resection was performed to prevent potential cardia or pyloric stenosis. The lesion was excised using ultrasonic knives with a margin of about 20 mm, and the stomach wall defect was repaired using laparoscopic linear cutting stapler or by hand suturing. The resected tumor was placed into a specimen bag and removed through a median upper abdominal incision.

### Postoperative management

All patients received gastrointestinal decompression and were fasted for at least 72 h. Proton pump inhibitor was given intravenously for at least 3 days, and prophylactic antibiotics and parenteral nutrition support were routinely used for 2–3 days. If the gastrointestinal function was restored and there was no gastrointestinal bleeding, the diet was gradually transitioned from liquid to the semi-liquid food. In addition, abdominal signs and symptoms were closely observed during the whole postoperative period.

### Pathological evaluation

The resected tumors were placed in 10% formalin solution and were sent to the pathology department for pathological evaluation. The characteristics of gross specimens including gross appearance, tumor size and ulceration were described. Hematoxylin and eosin staining was performed in all specimen. Imunohistochemical staining including S-100, SOX10, Dog-1, CD117, CD34, SMA and Desmin were conducive to the differential diagnosis with other gastric submucosal tumors. The lesion positive for S-100 but negative for CD117, DOG-1,SMA and Desmin was diagnosed as GS.

### Follow-up

Patients were scheduled for the first endoscopic to observe the wound healing in 3–6 months after operation, and thereafter abdominal CT and endoscopy were performed annually for 60 months to evaluate the metastasis, recurrence or any residual tumor. No tumor recurrence and metastasis in 36 months indicated that laparoscopic surgery had favorable long-term results.

### Statistical analysis

Statistical analysis was conducted using SPSS 17.0 software (SPSS, Chicago, Ill). Continuous values were presented as the mean ± standard deviation, while categorical data were expressed as percentages and numbers.

## Results

### Clinical findings

Four hundred thirty-two patients were pathological diagnosed as schwannoma in our hospital between January 2014 and December 2020 and only 13 GS patients. According to the inclusion criteria, a total of 10 patient with GS were hereby included, with the symptoms and demographic data summarized in Table 1. There were 7 females and 3 males, and the male-to-female ratio was 1:2.33. The mean age of patients was 51.6 ± 7.3 years. Upper abdominal pain was the most common symptom and was observed in 7 patients (70%). Meanwhile, 2 patients (20%) were asymptomatic, and 1 suffered from upper gastrointestinal hemorrhage. All patients had no history of type 1 or type 2 neurofibromatosis syndrome.

**Table 1:** Clinical characteristics of 10 patients with gastric schwannoma

Case	Gender	Age (year)	Clinical presentation	Location	Preoperative Diagnosis	Operation	Size (mm)	Follow up (month)	Outcome
1	Female	46	upper abdominal pain with belching	Gastric body	GIST	Laparoscopic wedge resection	32	105	alive
2	Female	47	asymptomatic	Gastric body	GIST	Laparoscopic wedge resection	70	83	alive
3	Female	63	upper abdominal pain	Gastric fundus	GIST	Laparoscopic local excision	60	53	alive
4	Female	55	upper abdominal pain	Gastric body	GIST	Laparoscopic wedge resection	60	48	alive
5	Female	48	upper abdominal pain	Gastric antrum	GIST	Laparoscopic wedge resection	38	45	alive
6	Female	45	upper abdominal pain	Gastric antrum	GIST	Laparoscopic local excision	60	31	alive
7	Male	50	upper abdominal pain	Gastric body	Protruding lesions	Laparoscopic wedge resection	35	25	alive
8	Male	56	gastrointestinal hemorrhage	Gastric body	GIST	Laparoscopic wedge resection	35	22	alive
9	Male	63	upper abdominal pain	Gastric body	GIST	Laparoscopic wedge resection	55	21	alive
10	Female	43	asymptomatic	Gastric body	Protruding lesions	Laparoscopic local excision	55	18	alive

*GIST* Gastrointestinal Stromal Tumor

### Laboratory examinations and imaging findings

Laboratory examinations were conducted in all patients, acquiring unremarkable results of the complete blood count, kidney function, liver function, coagulation function test and plasma electrolyte. Besides, tumor markers, including CEA, CA19-9, AFP and CA50 were all normal.

**Fig. 2:**
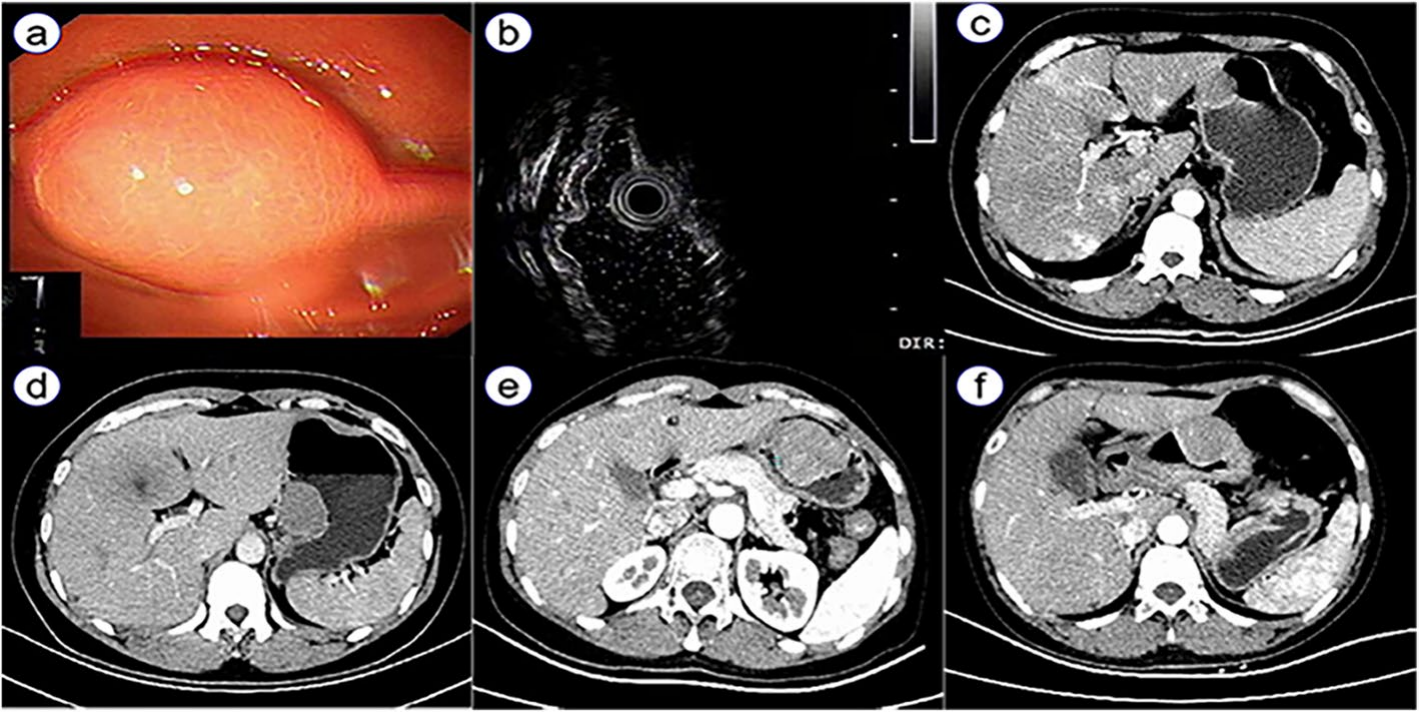
The diagnostic of characteristic imaging of gastric schwannomas. **a** endoscopy present as a smooth, round, elevated submucosal masses; **b** hypoechoic muscularis propria lesions with clear margin; **c** the mass with slight uniform enhancement CT; **d** another mass with slight uniform enhancement CT; **e** the mass with moderate uniform enhancement CT; **f** another mass with moderate uniform enhancement CT

CT was performed in all patients before surgery and a well-defined round soft tissue tumor was observed in all cases. Among the 10 patients, 8 had moderate uniform enhancement, and 2 had slight uniform enhancement. Perigastric lymph nodes were observed in 6 (60%) patients. Additionally, 8 were diagnosed as GIST, whereas 2 were diagnosed as gastric protruding lesions in CT scan. Preoperative gastroscopy was performed in all cases, which indicated submucosal tumors, of which, 5 cases were suspected as GIST. Only one patient exhibited mucosal ulceration. Besides, endoscopic ultrasonography was performed in 6 patients, which showed homogenous and hypoechoic muscularis propria lesions with clear margin, lack of cystic change and calcification. The diagnostic of characteristic imaging is shown in Fig. 2.

### Surgical results

Herein, 8 patients were preoperatively diagnosed as GIST, and 2 as gastric protruding lesions. Surgical procedures included laparoscopic gastric wedge-resection (7 cases) and laparoscopic gastric local resection (3 cases). The most common site was gastric body (7/10, 70%), 2 in antrum and 1 in fundus. All patients achieved complete resection. The mean operation time was 112.6 ± 34.3 min, and the postoperative hospital stay of patients was 13.8 ± 5.1 days. Postoperative gastroplegia occurred in 2 patients with tumors located in less curvature, which may caused by injury of the trunk of the vagus nerve during omental and tumor resection. Although 2 patients recovered from gastrointestinal decompression, fasting and water deprivation and parenteral nutrition, it also increased the mean length of hospital stay. There was no mortality.

### Pathologic and immunohistochemical findings

**Fig. 3:**
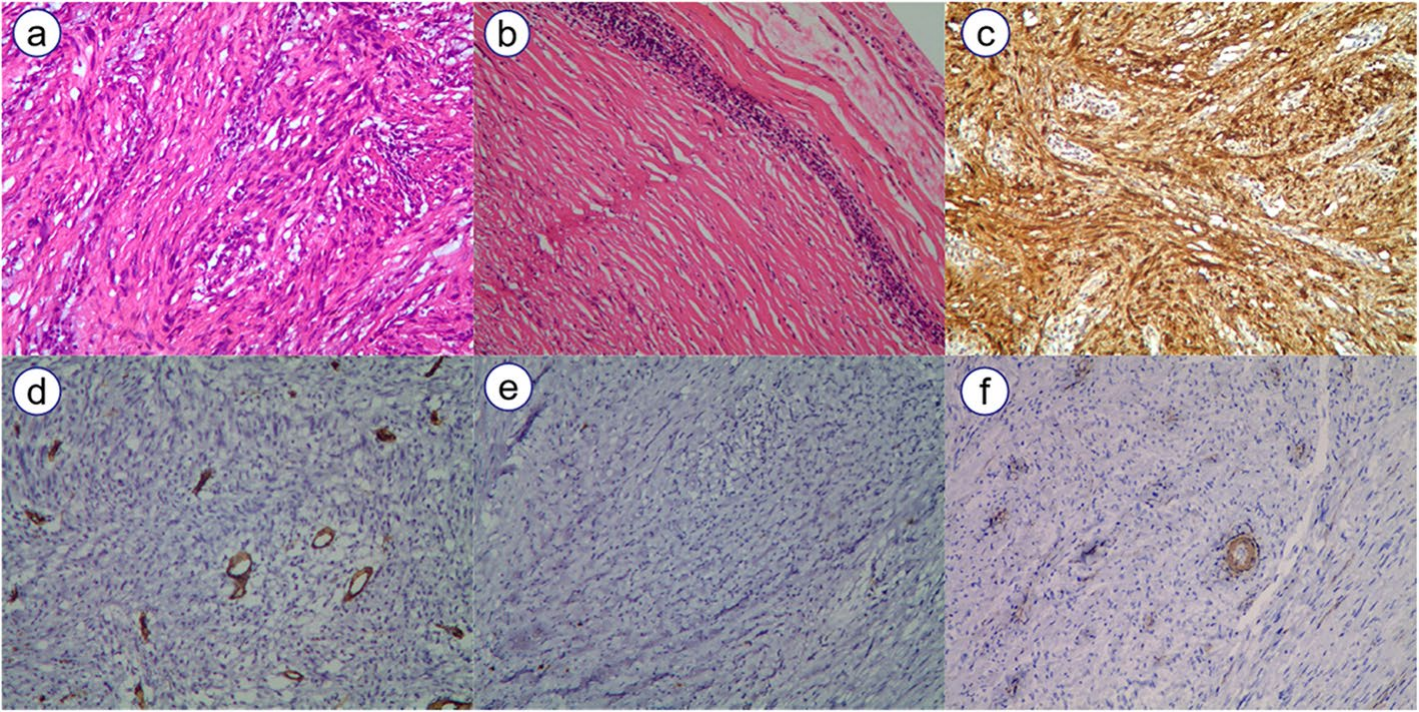
**a** tumors were composed of spindle cells; **b** lymphocytic peritumoral cuff. **c** S-100 strong positive; **d** CD34 vascular positive; **e** CD117 negative; **f** SMA negative ( 10 × 10 magnification)

All patients were pathologically diagnosed as GS. The mass color was greyish-white in 9 patients and greyish-yellow in 1 case. All tumors had distinct borders, and the mean size was 50.0 ± 13.6 mm. Histology showed that all tumors were composed of spindle cells and arranged in an a microtrabecular or microfascicular pattern with lymphocyte infiltration. A lymphocytic peritumoral cuff was present in all (100%) cases. Immunohistochemical staining was performed for all patients. All cases showed S-100 protein positive, and SOX10 was also detected positive in 5 patients. Most cases (7 cases) were negative for CD34, while 3 cases were vascular positive. DOG-1, CD117, SMA and Desmin were negative in all cases (Fig. 3). The mean Ki67 labeling index was 2.8% ± 1.9%.

### Follow up results

As of December 2022, all 10 patients were followed up for 45.1 ± 29.0 months without residue tumor, significant metastasis or recurrence. Only one patient developed upper abdominal pain accompanied by black stools 4 months after surgery, and gastroscopy indicated gastric antrum ulcers and gastric retention. The patient was recovered from blood transfusion, fasting, acid suppression and nutritional support therapy.

## Discussion

GS is a rare sub-type of gastric mesenchymal tumor, also a slowly-growing encapsulated tumor originating from schwann cells. GS has also been reported as a female-predominant disease with a male to female ratio of 1:2 or ever higher, which usually occurs between the ages of 40 to 60 years [3]. Most GS patients are asymptomatic and often discovered incidentally during unrelated medical procedures or physical examination [12]. A small proportion of GS patients may present with non-specific symptoms including stomach discomfort or upper abdominal pain, gastrointestinal bleeding and palpable masses [3, 13]. In this study, the clinical characteristics of the patients were consistent with previous study.

The differential diagnosis of gastric submucosal tumors is difficult before surgery, especially in distinguishing GS from GIST. Enhanced CT and endoscopy are the most valuable preoperative examinations. On CT examination, GS often present as a well-defined round or oval tumor under the gastric mucosa with an exogenous or mixed growth mode and moderate homogeneous enhancement [14]. GS frequently occurs with enlarged reactive inflammatory perigastric lymph nodes. Previous studies showed that the incidence of peritumoral lymph nodes ranged from 47.4% to 81% [14, 15]. Perigastric lymph nodes are CT feature for GS. Herein, perigastric lymph nodes were found in 6 (60%) patients, which were consistent with previous reports. Besides, GS rarely presents degenerative changes, whereas GIST can be easily developed to hemorrhage, necrosis and cystic change [16]. The presence of necrosis is a significant CT feature suggestive of GIST [17].

On endoscopy, GS usually presents as smooth, round and elevated submucosal masses, with or without mucosal ulcer, making it different from GIST [18]. Traditional endoscopic mucosal biopsy often shows false negative results. In the present study, none of the 10 patients were preoperatively diagnosed as GS by endoscopic biopsies, and all biopsy pathology suggested chronic inflammation. Generally, EUS can provide significant information about the tumor size, location, define the origin level, depth of invasion and echogenicity. On EUS evaluation, the typical features of GS are heterogeneous hypoechogenicity, rounded submucosal mass with well-defined margin, lack of calcification and cystic change and mostly originating from the muscularis propria [19]. Endoscopic ultrasound-guided fine-needle aspiration (EUS-FNA) is an significant method for the diagnosis of gastric submucosal tumors with a diagnostic accuracy rate of 43.3% to 52% [20, 21]. However, EUS-FNA increases the risk of tumor rupture and spread, thereby leading to poor prognosis. Both in this study and previous research [22], most of GS patients were diagnosed as GIST, and EUS-FNA were not recommend routine for primary resectable GIST according to National Comprehensive Cancer Network guidelines [23]. Therefore EUS-FNA was not routinely performed in this study.We recommend abdominal enhanced CT and EUS as routine examinations for GS.

The definitive diagnosis of GS is determined by immunohistochemical and pathological examination. GS is typically positive for S-100 and occasionally locally positive CD34, but negative for CD117, DOG-1, SMA and Desmin [13, 24, 25]. High nuclear atypia, lower S-100 expression, mitotic over 15/50 HPFs and higher Ki-67 expression are characteristics of malignant GS [26]. Pathologically, the tumor is composed of spindle-shaped cells with a lymphocytic peritumoral cuff [27]. In this study, S-100 was positive in all cases, and vascular positive for CD34 in 3 case. All cases were negative for CD117, DOG-1, SMA and Desmin and had lymphocytic peritumoral cuff. These results were consistent with previous literature.

Because of difficulties to distinguish GS from other gastric submucosal tumor, the presence of lesions imposes a psychological burden to patients, and many patients request for surgical resection [28]. Laparoscopic gastrectomy is a treatment option for GS, including wedge resection, local resection, subtotal or total resection, among which, laparoscopic gastric wedge resection has been frequently performed. Lymphadenectomy is not routinely performed. Malignant GS is mainly based on immunohistochemical features and is difficult to be diagnosed preoperatively, so the treatment strategy for GS is complete resection with preservation of gastric function regardless of the tumor is malignant or benign [26, 29]. Laparoscopic surgery for GS is associated with shorter postoperative hospital stay, less blood loss and lower incidence of postoperative complications compared with open surgery [22]. In the case of choosing the surgery type, tumor size and location, the relationship with surrounding organs and the surgical skill of surgeon should all be taken into serious consideration. Large lesions or lesions located in the pylorus or cardia are not suitable for gastric wedge resection due to potential stenosis, and local resection can be used in these patients.

Endoscopic resection has been widely regarded as a safe and effective surgery for GS [30–33]. Zhai et al. [31] suggested that lesions larger than 50 mm were not suitable for endoscopic resection, and endoscopic resection may be a more feasible option for selected patients with tumors smaller than 30 mm. Zhou et al. [32] obtained the accordant results. In their research, four lesion sizes were larger than 30 mm and should be divided into 2 to 4 pieces in the stomach to allow retrieval from the oral. Besides, bleeding and perforation are more likely to occur in patient with larger tumor. Thus, previous study recommended surgery for tumors larger than 30 mm [33]. Herein, all tumors were larger than 30 mm and were complete resected by laparoscopic surgery without recurrence or residue during a mean follow-up of 45.1 months. It was believed that laparoscopic gastric resection may be the most preferred choice for patients with GS larger than 30 mm.

Additionally, the postoperative prognosis for benign GS was excellent with 5-year overall survival rate was over 95% [3]. In a systemic review, there was no metastasis or recurrence in 137 GS patients during 1 to 336 months follow-up [34]. Malignant GS patients had a poor prognosis with an average 5-year survival rate of 23%. Most patients suffered from rapidly progressive diseases. In the present study, all patients were benign GS, and no metastasis and recurrence occurred during the follow-up period.

However, this study still has some limitations. First, due to the low incidence, the sample size of this study was small, and all tumors were larger than 30 mm; Second, the study is a retrospective case series study and contains some deviations. Finally, laparoscopy surgery was not compared with open surgery or endoscopic surgery for GS.

## Conclusion

In conclusion, 10 cases of GS with a diameter over 30 mm were hereby reported. This tumor is rare gastric submucosal tumor and is often preoperatively misdiagnosed as GIST. Laparoscopic resection including wedge resection, local resection is a safe and effective method for the management of GS with a diameter over 30 mm and has favorable long-term follow-up outcomes. Laparoscopic resection may be considered as the first-choice treatment for patients with GS larger than 30 mm.

## Data Availability

The datasets supporting the conclusions of this article are available from the corresponding author on reasonable request.
